# Acupuncture Treatment of Lateral Elbow Pain: A Nonrandomized Pilot Study

**DOI:** 10.1155/2016/8182071

**Published:** 2016-02-24

**Authors:** Yan-Song Liu, Marcus Gadau, Guo-Xue Zhang, Hao Liu, Fu-Chun Wang, Christopher Zaslawski, Tie Li, Yuan-Sheng Tan, Christine Berle, Wei-Hong Li, Sergio Bangrazi, Stefano Liguori, Shi-Ping Zhang

**Affiliations:** ^1^Changchun University of Traditional Chinese Medicine, Changchun 130117, Jilin, China; ^2^School of Chinese Medicine, Hong Kong Baptist University, Kowloon Tong, Hong Kong; ^3^College of Traditional Chinese Medicine, University of Technology Sydney, Sydney, NSW 2007, Australia; ^4^World Federation of Acupuncture and Moxibustion Societies, Beijing 100061, China; ^5^Istituto Paracelso, Rome 00153, Italy

## Abstract

In planning for a large-scale multicenter trial to evaluate the effect of acupuncture for the treatment of lateral elbow pain, a pilot study was conducted. This was a prospective, investigator- and patient-blinded, nonrandomized, placebo controlled trial. Subjects were evaluated at baseline, before fourth, seventh, and ninth treatment, and at a two-week posttreatment follow-up. The treatment group received unilateral acupuncture at LI 10 and LI 11 at the affected side with manual needle manipulation; the control group received sham-laser acupuncture at the same acupoints. Measures included (i) disabilities of the arm, shoulder, and hand (DASH) questionnaire, (ii) pain-free grip strength (PFGS), and (iii) a visual analogue scale (VAS) for pain. Significant differences in DASH score, PFGS, and VAS between treatment and control group were found at the ninth treatment (*n* = 20 for each group, *P* < 0.05). Only DASH showed significant differences compared to the control for all the measurement time points after treatment commenced and appears to be a sensitive and appropriate primary outcome measure for the future multisite trial. Results from this pilot study provided relevant information about treatment efficacy, credibility of control treatment, and sensitivity of different outcome measures for the planning of the future trial.

## 1. Introduction

*Background*. Acupuncture has been used for the treatment of many musculoskeletal conditions, including lateral elbow pain (LEP), or tennis elbow. However, a high level of evidence supporting the use of acupuncture treatment for LEP is lacking [1–4]. In our latest review of the topic, we have found some evidence suggesting that acupuncture might be more effective than sham acupuncture or ultrasound treatment. However, it was also found that there are many limitations associated with the previous studies, such as a lack of standardization of outcome measures, no clear statement of primary outcome measure, and much variation in the administration of acupuncture and in the selection of a control treatment [2].

We have planned to conduct a four-site trial in four countries/regions to determine the efficacy of acupuncture treatment for LEP, with the goal to minimize limitations of previous studies. Multisite randomized controlled trials (RCTs) are commonly used in pharmaceutical studies, as they have the ability to capture larger sample sizes and their findings are more generalizable than the findings of single-site RCTs. Moreover, multisite RCTs can facilitate the adaptation of best practice in the selection of treatment protocols and outcome measures, as there are a larger number of experienced researchers involved in the design and execution of the trial.

The selection of a treatment regime is challenging as positive results have been reported for manual needling, electroacupuncture, and manual acupuncture combined with moxibustion [5–7]. Within manual needling there are also many variations in the needling technique. Similarly, different outcome measures have been used to evaluate the efficacy of treatment for LEP, including pain, functional impairment, and grip strength [5, 8, 9]. In view of the need to establish an easy-to-replicate method of acupuncture treatment for LEP, it was decided to trial a simple manual needle manipulation technique that has been used by Fu-Chun Wang, one of the experts in our study group. In our planning meeting it was apparent that a pilot study was needed to assess the adequacy of the initial treatment protocols and outcome measures.

*Objectives*. Through the pilot study it was hoped to gain some information regarding the following: first, we wanted to observe the efficacy of the selected acupuncture treatment protocol. Second, we wanted to know which outcome measure was sensitive and could be used in the future multisite trial as the primary outcome measure. Third, we wanted to gather information on the standard deviation of the primary outcome measure in the studied population, which would be crucial for determining the sample size of the multisite trial. Finally, we wanted to determine the credibility of the control intervention, which was a sham-laser treatment (deceptive nonactive laser treatment). To this end, a two-arm, nonrandomized study using sham-laser treatment as control was undertaken at one of the study sites. Randomization of subjects was not performed due to a time constraint because the laser unit was not available at the beginning of the trial.

## 2. Methods

### 2.1. Trial Design

This study was designed to be a care-provider-, assessor-, and patient-blinded, nonrandomized (group allocation in two blocks based on the time the subject entered screening), placebo controlled (sham-laser), parallel arm trial to evaluate the efficacy of acupuncture in the treatment of LEP. This trial specifically served as a pilot study to test the efficacy of the treatment protocol and the suitability and sensitivity of the outcome measures and to serve as a basis to determine the sample size of a much larger, multicentered, international RCT (tennis elbow acupuncture international study, China, Hong Kong, Australia, and Italy: TEAISCHAI).

### 2.2. Subjects

This study took place from February 2012 until December 2012 and the protocol was approved by the Changchun University of Traditional Chinese Medicine and adhered to the Declaration of Helsinki [10]. The study was advertised in the press and subjects were instructed to contact the research team via telephone. Prescreening was carried out by means of a semistructured telephone interview. Subjects were mainly referred from the outpatient department of the Changchun University of Traditional Chinese Medicine Hospital. As a result of this, 53 subjects who appeared to be suitable for enrolment were invited for screening. After careful screening through history taking and clinical examination for inclusion and exclusion criteria, 41 subjects were selected and enrolled in the trial after they had given informed consent. Inclusion criteria were adult population from 18 to 70 years with a history of unilateral chronic elbow pain for over three months. We excluded subjects who had a history of central or peripheral nervous system disease, inflammatory rheumatic diseases, radial nerve entrapment, radioulnar or radiohumeral osteoarthritis, gout, earlier episodes of lateral elbow pain treated surgically, earlier episodes of lateral elbow pain treated with acupuncture within the previous six months, or acupuncture treatment for any problems within the previous week. Subjects with pregnancy or needle phobia were also excluded. The diagnostic criteria for lateral epicondylitis were the following: (i) typical history of lateral elbow pain, (ii) tenderness and pressure pain on the radial epicondyle of the humerus (insertion of common extensor tendon), and (iii) aggravation of pain during extension of the wrist against resistance.

### 2.3. Interventions

#### 2.3.1. Treatment Intervention

*Rationale*. We based the acupuncture point selections on Traditional Chinese Medicine meridian theory [11], frequently recommended points to treat LEP [12], and the consensus found during an initial meeting of all centers of the TEAISCHAI study. Special attention was given to the needle stimulation technique, which was based on the “green dragon wags its tail” method originating from the Zhen Jiu Da Quan (Complete Book of Acupuncture-Moxibustion) [13], as well as over 30 years of clinical experience from Fu-Chun Wang, one of our experts on needling techniques.

Standardized interventions were developed for both treatment and control groups. Interventions did not vary within one group and all subjects receive the same intervention at all treatment sessions for their group. Practitioners were required to apply standardized intervention protocols to subjects, dependent on the participant's group identity.

*Needling Details*. Two acupoints LI 11 (Quchi) and LI 10 (Shousanli) were needled with single-use, stainless steel, sterile, filiform 0.30 mm × 40 mm Hua Tuo needles unilaterally on the effected side per subject per treatment session. They were located according to the “WHO Standard Acupuncture Point Locations in the Western Pacific Region” (World Health Organization 2008) [14]. LI 11 (Quchi) was needled first, perpendicular insertion up to 1.5 cun was performed, and then the needle was withdrawn to one-cun depth. Then LI 10 (Shousanli) was needled; oblique insertion at 45° pointing towards the elbow was performed to a depth of one cun. Needling sensation (Deqi) was sought on both acupoints. Muscle twitch was recorded on subject's daily reporting sheet if achieved. Manual needle manipulation was used. For LI 11 (Quchi) the practitioner held the needle on the end of the handle and bent it 45° left and right with a speed of one Hz for two minutes or according to participant's tolerance. This procedure was then repeated for LI 10 (Shousanli) and was again repeated for both acupoints prior to removing the needles. Needles were retained in situ for 25 minutes.

*Treatment Regimen*. For both, the treatment and the control group, each intervention session lasted 25 minutes. Subjects were asked to attend three sessions per week (Monday, Wednesday, and Friday) for three weeks, totaling nine sessions during the entire trial period.

*Other Components of Treatment*. There were no other interventions administered to either group. However, subjects using analgesics, NSAIDs, or exercise programs within the last two weeks prior to commencing the trial were requested to maintain their current regime but needed to keep a record in their diary.

Treatments were undertaken at the outpatient department of Changchun University of Traditional Chinese Medicine Hospital. Video and hard copy instructions were prepared to train practitioners to ensure standardization of interventions. Subjects were well informed about the study interventions.

*Practitioner Background*. The practitioner delivering the interventions was a qualified acupuncturist with a minimum of seven-year clinical practice. The clinician was competent and familiar with the relative treatment protocol.

#### 2.3.2. Control Intervention

*Rationale*. Placebo acupuncture control interventions (sham acupuncture) have not yet been standardized and there is no agreed consensus within the scientific community on which sham acupuncture method should be used [15]. We therefore refrained from inserting needles, which might cause a microinjury and might trigger a cascade of physiologic reactions, and used an inactive laser unit instead.

*Laser Details*. The inactive laser unit was rested lightly on the skin at the same acupoints as the treatment group. Firstly, the subject was told that LI 11 (Quchi) was treated for two minutes, after which the subject was asked to rest for ten minutes, and then LI 10 (Shousanli) was treated for two minutes, after which the subject was asked again to rest for ten minutes. The entire procedure took 24 minutes.

The laser unit (H6 N6 Laser Acupuncture Instrument, Model HY369-B) had the laser diode replaced by an LED light and a sound upon switching it on and off indicated functionality. Participants were warned about the “harmfulness” of the laser to the eyes, and the laser was held away from the participants' eyes at all times. The participants as well as the practitioner were asked to wear special laser-protection glasses during the course of the intervention. The intervention regimen (duration, number, and frequency of intervention sessions) was the same as for the treatment intervention.

A credibility rating scale [16] was used to identify the credibility and adequacy of the control intervention. After allocation but prior to the first intervention, the intervention's credibility rating scale (CRS) was administered to each subject regardless of group identity. Participants were asked to choose the most suitable answer for four statements on a five-point scale. The four statements were as follows: (i) “My illness will improve considerably”; (ii) “I will be able to better cope with my illness”; (iii) “The condition's symptoms will disappear”; (iv) “My mental state will improve because of the intervention.” Zero points on the CRS indicated that subjects expected no efficacy, one point indicated little efficacy, two points indicated medium efficacy, three points indicated good efficacy, and four points indicated that subjects expected to be fully cured due to the intervention that they would receive.

### 2.4. Outcome Parameters

Prior to initial interventions, (i) demographic data (gender, age, and duration of condition), (ii) disabilities of the arm shoulder and hand (DASH) score, (iii) pain-free grip strength (PFGS), and (iv) pain, using a visual analogue scale (VAS), were obtained.

During the nine intervention visits DASH, PFGS, and VAS were assessed four times: prior to the first, fourth, seventh, and ninth visit. During a follow-up visit two weeks after the last (ninth) intervention, the outcome measures of DASH, PFGS, and VAS were reassessed at which point the study ended. The outcome assessor was blinded with respect to the subject's group identity and had no knowledge which intervention technique was applied.

The DASH questionnaire was used to assess functional impairment of the elbow [17]. Subjects were asked to answer all 30 questions of the main module, not including the two optional models.

Muscle strength (PFGS) was assessed using a hand held dynamometer (model J00105 JAMAR), while the subject was seated on an armchair with the forearms supported by the arm of the chair and the wrists just over the end of the chair, forearms semipronated, wrists in neutral position, and thumbs facing upwards. Three measurements were taken for both arms with ten to twenty seconds rest in between each measurement. The mean of the three measurements was used for analysis. Subjects were asked to squeeze as long and as tight as possible but to stop squeezing if they felt pain. PFGS is considered a valid and reliable outcome measure for LEP [18].

Pain assessment was conducted by using a pain-monitoring questionnaire consisting of a 100 mm long nonsegmented line (VAS). Subjects were asked to indicate with an “*x*” across a vertical line the level indicating their pain at rest, in motion, and during exertion, starting from 0 mm, no pain at all, to 100 mm, excruciating pain. VAS has been frequently used in pain evaluation studies and has been found to be a reliable and valid measurement with the LEP population [5, 19, 20].

Adverse events were to be noted and reported by the practitioner.

### 2.5. Allocation and Blinding

Subjects were allocated to either treatment or control group based on when they entered the screening phase. During the first screening phase twenty-one subjects were enrolled in the treatment group. The second screening phase was terminated after 20 subjects enrolled in the control group. Subjects, care-providers, and assessors were blinded with respect to the efficacy of the control intervention. That is, even though subjects obviously knew that they received laser treatment instead of acupuncture, they were not aware that the laser unit was inactive. Likewise, the care-provider and assessor were not aware that the laser diode was replaced with an LED light. Guo-Xue Zhang screened and enrolled the subjects and provided treatment. Hao Liu conducted the outcome assessments; both were blinded. Yan-Song Liu was not blinded, coordinated the trial, and assigned which of the two blocks would receive which intervention.

### 2.6. Statistical Analysis

Statistical analysis was performed using the statistical package for the social sciences (SPSS) version 21. Distribution of data was tested by (a) numerical methods and (b) graphical methods. Numerical methods included (i) assessment of *z*-scores for skewness and kurtosis and (ii) the Shapiro-Wilk's test for both groups. Visual methods included (i) visual inspection of histograms and (ii) visual inspection of normal Q-Q plots for both groups.

Mann-Whitney *U* tests were performed for baseline, credibility rating, and between-group analyses. Pairwise comparisons of DASH scores, PFGS, and VAS at rest, in motion, and during exertion were performed with a Bonferroni correction for multiple comparisons. To determine the difference between the two groups, we compared the mean differences relative to baseline instead of the actual mean values to compensate for baseline differences between the two groups. Distributions of scores for both groups were assessed by visual inspection. If distributions were of similar shape, medians were compared. If distributions were of dissimilar shape, mean ranks were compared.

For within-group analysis we used Friedman's two-way analysis of variance by ranks test comparing median DASH scores, PFGS, and VAS at rest, in motion, and during exertion at a given time point with the respective baseline value. Pairwise comparisons were performed with a Bonferroni correction for multiple comparisons.

## 3. Results

Normality tests showed that the majority of the data was not normally distributed and could not be transformed to meet assumptions for parametric tests; therefore nonparametric tests for data analysis were performed.

**Figure 1:**
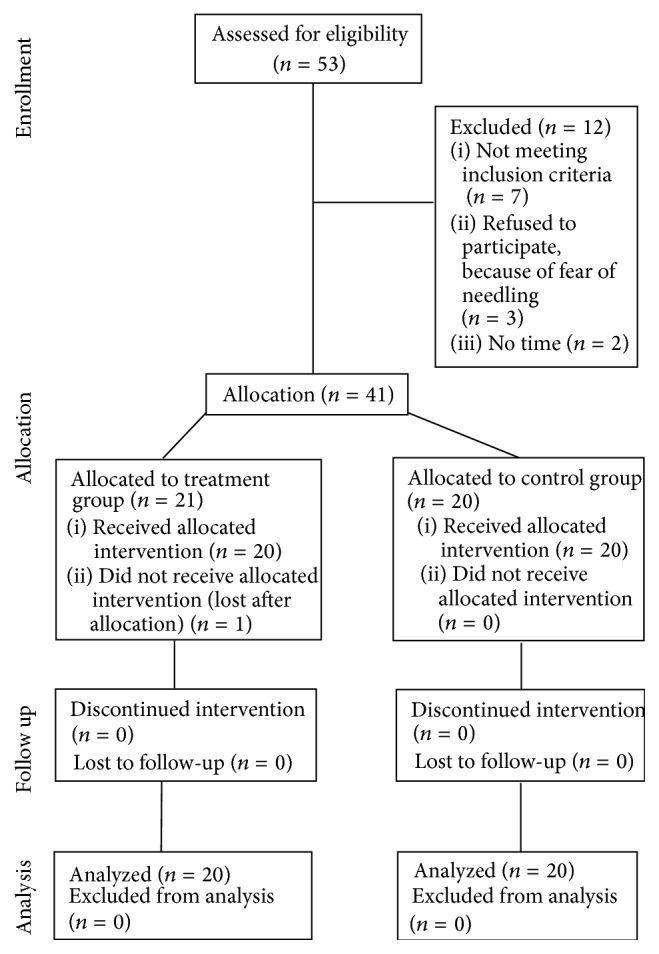
Flow chart trial participants.

Out of the 53 subjects initially assessed for screening, seven were excluded because they did not meet the inclusion and exclusion criteria, three refused to participate, because of fear of needling, and two refused after they were given detailed information about the trial, because they could not find enough time. Forty-one subjects entered the trial and were allocated to either treatment group (*n* = 21) or control group (*n* = 20). One subject did not attend the course of treatment and thus was not included in the analysis.  Figure 1 shows the details of the trial participants and their progress through the stages of the trial according to the CONSORT statement [21].

Baseline information of the subjects is reported in Table 1. Independent samples Mann-Whitney *U* tests showed that the two groups' demographic characteristics as well as baseline measurements of DASH, PFGS, and VAS were all similar, except the control group's duration of the condition (mean rank = 24.15) which was significantly higher than the treatment group's (mean rank = 16.85) (*P* = 0.047).

**Figure 2:**
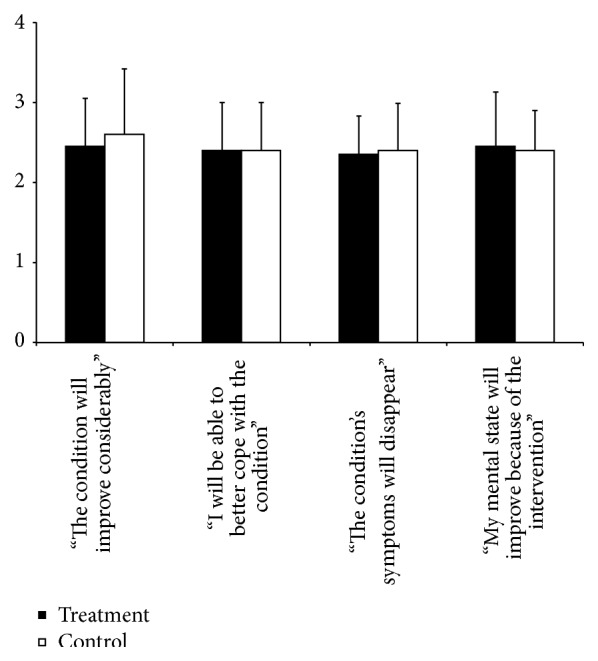
Credibility assessment comparing treatment and control interventions using a five-point rating scale (*n* = 20 for each group). Histograms display means, error bars show standard deviations (SD), and no significant difference between groups was found.

The results of the credibility rating scale are shown in Figure 2. No significant differences in efficacy expectancy between the two groups were found (e.g., improvement of condition expectancy, *P* = 0.71, Mann-Whitney *U* test). This suggests that participants rated the control intervention (sham-laser) as a highly credible form of treatment in this current setting.

There were significant within-group differences for all measurements in the acupuncture group when comparing the last treatment (ninth treatment) and the two-week follow-up visit with the baseline (Friedman's test) (see Table 2). For DASH, VAS at rest, and VAS during exertion, statistically significant differences were observed as early as the seventh treatment visit (Table 2).

We found significant differences in the control group when comparing baseline to the ninth and the two-week follow-up visit for DASH scores and for VAS during exertion. Significant difference was also found between baseline and the seventh visit for PFGS measurements. For other time points of DASH, PFGS, and VAS during exertion, no significant differences were found. There were also no significant differences between baseline and any time point for VAS at rest and VAS in motion in the control group (Table 2).

**Figure 3:**
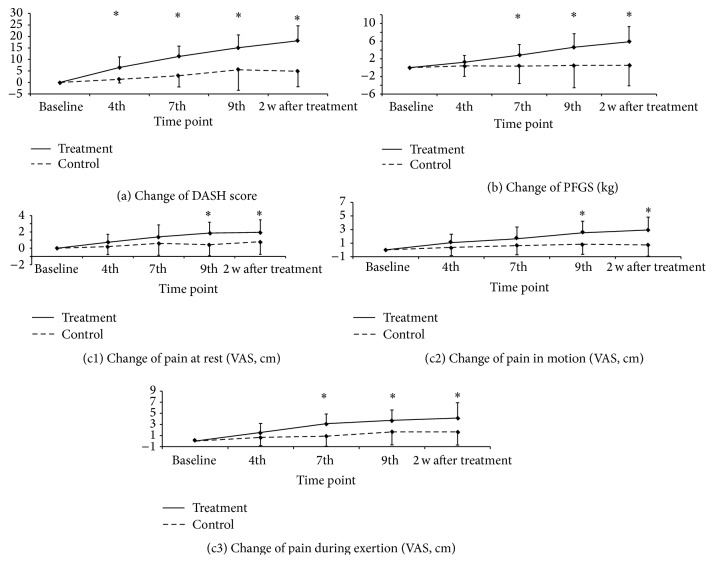
DASH and PFGS changes over time. Line graphs showing changes of the two groups from baseline throughout the trial period for (a) disabilities of the arm, shoulder, and hand (DASH) score (functional impairment) and (b) pain-free grip strength (PFGS) (*n* = 20 for each group). Error bars show standard deviations (SD); *∗*: statistically significant difference (*P* < 0.05) between the two groups at a given time point. (c1)–(c3) VAS changes over time. Line graphs showing changes of the two groups from baseline throughout the trial period for (c1) pain at rest, (c2) pain in motion, and (c3) pain during exertion on visual analogue scales (VAS) (*n* = 20 for each group). Error bars show standard deviations (SD); *∗*: statistically significant difference (*P* < 0.05) between the two groups at a given time point.

Mann-Whitney *U* tests were run to determine if there were differences in improvement from the baseline value at a given time point between the two groups. We found significant differences in functional impairment (DASH scores) already from the fourth visit to the follow-up visit. For muscle strength (PFGS) significant differences were observed from the seventh visit onwards. Pain at rest (VAS rest) and pain in motion (VAS motion) showed significant differences between the two groups from the last (ninth) treatment visit onwards. Pain during exertion (VAS exertion) significantly improved from the seventh visit onwards (Figures 3(a), 3(b), 3(c1), 3(c2), and 3(c3)).

No adverse events were reported.

## 4. Discussion

The present study was designed to serve as a pilot for a multisite international trial to evaluate the effectiveness of acupuncture treatment for tennis elbow. We found that the acupuncture treatment was efficacious compared to sham-laser treatment. At the end of the treatment and at two-week posttreatment, subjects in the acupuncture group showed significant differences in all outcome measures compared to the control group. These pilot results suggest that the manual acupuncture procedure is efficacious and can be used for a large multisite trial. Furthermore, we found that the control treatment was as credible as the acupuncture treatment to the subjects, although the procedure of sham-laser treatment was distinctly different from manual acupuncture. Similar credibility of sham-laser treatment has been reported previously [22].

Many different types of control have been used in acupuncture trials, but there has been no consensus as to which type is the best [23]. Recently, it has been argued that sham acupuncture is not an inert placebo [24], and there is evidence that some types of sham acupuncture produce physiological activity that are not due to a placebo effect [25, 26]. By using mock laser as control, we believe to have avoided the possible biological reactions to sham acupuncture. On the other hand, we believe that mock laser stimulation is just as valid as the usual sham acupuncture to be used as control, because it also has placebo effects. For example, we have previously found that both electroacupuncture and mock laser stimulation have similar efficacy in a large-scale clinical trial for neck pain [27].

Of the various outcome measures, only DASH showed significant difference compared to the control for all the measurement time points after the treatment commenced. Since DASH is a comprehensive evaluation of function rather than just pain, it appears to be a sensitive and appropriate measure to be used as the primary outcome measure for a future study. Thus, the DASH data from this pilot study will enable the estimation of an adequate sample size for a large-scale, multisite international trial.

A significant drawback of this study was the fact that the two groups of subjects entered into the trial at different time periods and that they were not randomized. This exposed our results to several confounding factors, such as cohort difference and seasonal changes. Therefore, when using our results for planning the future multisite trial, one should use the current data cautiously for sample size calculation. Another limitation of this study was that we only included subjects with unilateral elbow pain, which may lead to limitation in the generalizability of the results.

## 5. Conclusion

In conclusion, results from this pilot study have provided basic information about treatment efficacy, credibility of control treatment, and sensitivity of different outcome measures. This information has enabled us to plan for a large-scale, multisite trial to properly evaluate the effectiveness of acupuncture treatment for LEP.

## Figures and Tables

**Table 1:** Demographic data.

	Treatment group	Control group
Male (%)	11 (55)	8 (40)
Female (%)	9 (45)	12 (60)
Total	20	20
Age (mean ± SD)	42 ± 16.68	50.65 ± 7.27
Duration of condition (mean ± SD)	7.45 ± 6.09	9.45 ± 3.49^*∗*^
DASH (mean ± SD)	26.03 ± 7.61	21.79 ± 11.56
PFGS (mean ± SD)	16.54 ± 7.29	19.12 ± 9.15
VAS at rest (mean ± SD)	2.34 ± 1.28	1.65 ± 1.16
VAS in motion (mean ± SD)	3.96 ± 1.42	4.03 ± 1.31
VAS during exertion (mean ± SD)	6.62 ± 1.72	7.25 ± 1.62

*∗*: *P* = 0.047, significant difference between treatment and control group.

**Table 2:** Within-group comparison between baseline and different time points (*n* = 20 for each group).

Time point		DASH		PFGS		VAS at rest		VAS in motion		VAS in exertion	
		Treatment group	Control group	Treatment group	Control group	Treatment group	Control group	Treatment group	Control group	Treatment group	Control group
Baseline	Median (min.–max.)	24.58 (10.83–35.83)	18.33 (9.17–43.33)	16.55 (7.10–31.70)	17.23 (8.02–38.57)	2.55 (0.10–6.00)	1.45 (0.00–4.30)	3.65 (2.00–8.00)	4.15 (1.60–6.70)	6.70 (3.50–9.50)	7.35 (3.20–10.00)

4th treatment	Median (min.–max.)	19.16 (10.82–34.17)	16.66 (6.67–39.70)	17.84 (8.30–32.90)	16.50 (8.73–38.00)	1.45 (0.00–4.70)	1.05 (0.00–4.70)	2.95 (0.00–6.70)	3.20 (1.50–6.50)	4.85 (2.00–8.50)	6.35 (2.60–10.00)
Comparison to baseline (*n* = 20)	Median difference	*P* = 0.58	*P* = 0.80	*P* = 1.00	*P* = 0.74	*P* = 0.57	*P* = 1.00	*P* = 0.19	*P* = 1.00	*P* = 1.00	*P* = 1.00

7th treatment	Median (min.–max.)	13.34 (8.33–24.17)	17.08 (6.67–32.50)	19.45 (11.20–34.50)	17.48 (8.78–37.87)	0.55 (0.00–3.40)	0.90 (0.00–3.60)	1.75 (0.20–6.30)	2.90 (1.50–6.90)	3.15 (0.80–7.40)	6.20 (2.60–9.50)
Comparison to baseline (*n* = 20)	Median difference	*P* < 0.001^*∗*^	*P* = 0.64	*P* = 0.093	*P* = 0.07	*P* < 0.001^*∗*^	*P* = 0.40	*P* = 0.51	*P* = 1.00	*P* < 0.001^*∗*^	*P* = 1.00

9th treatment	Median (min.–max.)	10.83 (6.67–21.67)	17.08 (4.17–31.67)	20.55 (12.50–35.60)	18.81 (8.73–36.90)	0.50 (0.00–2.10)	0.95 (0.00–4.50)	1.25 (0.00–4.10)	2.60 (1.50–6.50)	2.40 (0.80–7.00)	5.80 (2.00–9.60)
Comparison to baseline (*n* = 20)	Median difference	*P* < 0.001^*∗*^	*P* = 0.001^*∗*^	*P* = 0.001^*∗*^	*P* = 0.39	*P* < 0.001^*∗*^	*P* = 0.40	*P* < 0.001^*∗*^	*P* = 0.054	*P* < 0.001^*∗*^	*P* = 0.03^*∗*^

2 weeks after treatment	Median (min.–max.)	6.67 (2.50–21.67)	15.83 (3.33–31.67)	21.20 (12.00–37.50)	16.46 (8.00–39.87)	0.00 (0.00–2.70)	0.90 (0.00–1.70)	0.60 (0.00–3.40)	2.70 (0.80–7.40)	1.85 (0.30–8.40)	5.25 (1.30–9.60)
Comparison to baseline (*n* = 20)	Median difference	*P* < 0.001^*∗*^	*P* = 0.001^*∗*^	*P* = 0.001^*∗*^	*P* = 1.0	*P* < 0.001^*∗*^	*P* = 0.06	*P* < 0.001^*∗*^	*P* = 0.12	*P* < 0.001^*∗*^	*P* = 0.012^*∗*^

DASH score: disability of the arm, shoulder, and hand score; PFGS: pain-free grip strength; VAS: visual analogue scale. *∗*: statistically significant difference (*P* < 0.05) between medians.

## Abbreviations

CRS: Credibility rating scale
DASH questionnaire: Disabilities of the arm, shoulder, and hand questionnaire
LED: Light-emitting diode
LEP: Lateral elbow pain
PFGS: Pain-free grip strength
RCTs: Randomized controlled trials
TEAISCHAI: Tennis elbow acupuncture international study, China, Hong Kong, Australia, and Italy
VAS: Visual analogue scale
WHO: World Health Organization.
